# Deciphering the Unique MicroRNA Signature in Human Esophageal Adenocarcinoma

**DOI:** 10.1371/journal.pone.0064463

**Published:** 2013-05-28

**Authors:** Rama Saad, Zheng Chen, Shoumin Zhu, Peilin Jia, Zhongming Zhao, M. Kay Washington, Abbes Belkhiri, Wael El-Rifai

**Affiliations:** 1 Department of Surgery, Vanderbilt University Medical Center, Nashville, Tennessee, United States of America; 2 The Biotechnology Graduate Program, The American University in Cairo, Cairo, Egypt; 3 Department of General Surgery, The First Affiliated Hospital of Nanjing Medical University, Nanjing, China,; 4 Department of Biomedical Informatics, Vanderbilt University Medical Center, Nashville, Tennessee, United States of America; 5 Department Pathology, Vanderbilt University Medical Center, Nashville, Tennessee, United States of America; 6 Department of Cancer Biology, Vanderbilt University Medical Center, Nashville, Tennessee, United States of America; 7 Department of Veterans Affairs, Tennessee Valley Healthcare System, Nashville, Tennessee, United States of America; Peter MacCallum Cancer Centre, Australia

## Abstract

**Background and Methods:**

Esophageal adenocarcinoma (EAC) is characterized by a steep rise in incidence rates in the Western population. The unique miRNA signature that distinguishes EAC from other upper gastrointestinal cancers remains unclear. Herein, we performed a comprehensive microarray profiling for the specific miRNA signature associated with EAC. We validated this signature by qRT-PCR.

**Results:**

Microarray analysis showed that 21 miRNAs were consistently deregulated in EAC. miR-194, miR-192, miR-200a, miR-21, miR-203, miR-205, miR-133b, and miR-31 were selected for validation using 46 normal squamous (NS), 23 Barrett’s esophagus (BE), 17 Barrett’s high grade dysplasia (HGD), 34 EAC, 33 gastric adenocarcinoma (GC), and 45 normal gastric (NG) tissues. The qRT-PCR analysis indicated that 2 miRNAs (miR-21 and miR-133b) were deregulated in both EAC and GC, and 6 miRNAs (up-regulated: miR-194, miR-31, miR-192, and miR-200a; down-regulated: miR-203 and miR-205) in EAC, as compared to BE but not in GC, indicating their potential unique role in EAC. Our data showed that miR-194, miR-192, miR-21, and miR-31 were up-regulated in BE adjacent to HGD lesions relative to isolated BE samples. Analysis of clinicopathological features indicated that down-regulation of miR-203 is significantly associated with progression and tumor stages in EAC. Interestingly, the overexpression levels of miR-194, miR-200a, and miR-192 were significantly higher in early EAC stages, suggesting that these miRNAs may be involved in EAC tumor development rather than progression.

**Conclusion:**

Our findings demonstrate the presence of a unique miRNA signature for EAC. This may provide some clues for the distinct molecular features of EAC to be considered in future studies of the role of miRNAs in EAC and their utility as disease biomarkers.

## Introduction

Although upper gastrointestinal adenocarcinomas (UGCs), stomach and esophagus, are closely related in location and molecular profiles, they have distinct pathophysiological characteristics. These cancers are usually diagnosed at advanced stages where poor prognosis remains a common feature [1]. A sharp increase in the incidence of esophageal adenocarcinoma (EAC) has been observed over the past three decades [2]–[5]. Understanding the unique molecular mechanisms underlying the development and progression of EAC represents an indispensable step for the development of specific diagnostic and therapeutic measures of EAC [1], [6].

A critical step in the fight against cancer is to identify those key changes occurring in the early stages of tumor development. MicroRNAs (miRNAs) are 20–25 nucleotide sequences that negatively regulate gene expression through targeting the complementary mRNA or blocking its translation [7]. Recently, miRNAs have gained significant attention because of their ability to regulate multiple oncogene and tumor suppressor signaling pathways [8], [9]. This fact has placed miRNA in the center of the cancer signaling network. Interestingly, miRNAs are known to be highly tissue specific in expression and function [10], [11]. The aberrant miRNA expression has been highlighted in the initiation and progression of several cancers including UGCs [7], [12]–[14]. Although a number of reports pointed out the dysregulation of several miRNAs in EAC, the unique miRNA signature that can distinguish EAC from the closely located gastric cancer has not been reported. Moreover, the differential miRNA expression associated with EAC progression across different stages is not well known. In this study, we performed comprehensive miRNA profiling and validation to identify miRNAs that are deregulated consistently and uniquely in EAC. In addition, we studied miRNA expression across the different stages of EAC, and compared miRNA expression between isolated Barrett’s esophagus (BE) and BE adjacent to HGD.

## Materials and Methods

### Ethics Statement

De-identified human tissue samples were obtained from the archives of pathology at Vanderbilt University (Nashville, TN, USA) and from the National Cancer Institute Cooperative Human Tissue Network (CHTN). The use of specimens was approved by the Institutional Review Board at Vanderbilt University Medical Center. All patients provided written consent, and samples were collected after surgical resection. All tissue samples included in this study were collected from tissues that remained after the completion of diagnosis, and are otherwise discarded.

### Sample Collection and Total miRNA Extraction

The samples included 46 normal squamous (NS), 13 isolated Barrett’s esophagus (BE), 10 BE adjacent to HGD, 17 Barrett’s high-grade dysplasia (HGD), 34 EAC tissues including frozen and formalin-fixed paraffin-embedded (FFPE), and 78 (45 normal gastric; NG, 33 gastric adenocarcinoma; GC) frozen gastric tissue samples. All of the NS tissues samples were obtained from different individuals. 38 NS samples were matched either with EAC (n = 23), isolated BE (n = 6), BE adjacent to HGD (n = 3) or HGD (n = 6). Out of the 17 HGD tissues, 8 were matched with BE adjacent to HGD. The 45 NG tissue samples were obtained from 44 different individuals. The 33 GC tissue samples were obtained from 32 different individuals. 20 out of the 33 GC tissue samples were matched with NG tissues. 25 of the GC tissue samples are obtained from the antrum, whereas 4 samples were obtained from the gastro-esophageal junction (GEJ), 2 from the cardia, and 2 from the body. Histopathological diagnosis was verified based on H&E-stained sections. The adenocarcinomas ranged from well-differentiated to poorly-differentiated with a mix of intestinal- and diffuse-type tumors. The total RNA, including miRNA, was extracted using a miRNeasy kit (Qiagen, Germantown, MD, USA) following the manufacturer’s protocol. In the case of FFPE tissues, an initial deparaffinization step was carried out using D-limonene (Sigma-Aldrich, Saint Louis, MO, USA) prior to extraction. Quality control analysis demonstrated no difference in the miRNA expression for samples purified from frozen or FFPE tissues.

### miRNA microarray analysis

Microarray analysis was performed using two independent platforms: Agilent Human microRNA Microarray V2 (Agilent Technology, Santa Clara, CA, USA) and Exiqon miRCURY LNA™ microRNA Array, v.11.0– human (Exiqon Life Science, Woburn, MA, USA), following the manufacturers’ protocols. This approach was chosen to detect the most significant changes and minimize artifacts. For the initial discovery phase, we analyzed 3 EAC samples as compared to a pool of 3 tumor-adjacent histological normal tissue samples used as a common reference to minimize variations. Preliminary analysis of the Agilent miRNA microarray data was performed utilizing the Agilent Feature Extraction (AFE) program to obtain the miRNA expression signal status. Subsequent analysis was carried out by means of R language (http://www.r-project.org). Following data transformation, inter-array normalization was performed using the quantile normalization method. Data were log2 transformed and the log2 ratio for each miRNA was computed by subtracting the log2 value in the pooled control sample from the average log2 values in three cancer samples. We utilized the *limma* R package to process the Exiqon miRNA expression data. The following steps were executed sequentially: (1) background correction; (2) intra-array normalization using the loess method; (3) for replicate probes, the median value was calculated to represent the miRNA intensity value; and (4) quantile normalization to allow comparison among arrays. To identify the differentially expressed miRNAs between the cancer and normal groups, we performed a two-class un-paired comparison and the *p* values were adjusted using the false discovery rate (FDR) method [3]. The set of miRNAs that overlapped between the two platforms to display at least a 2-fold change (FC) difference between both normal and tumor tissues were selected for validation to ensure that the difference is biological.

### Quantitative Real-Time PCR (qRT-PCR)

cDNA was synthesized by means of a 3-step protocol that includes poly(A) tail synthesis, followed by annealing of a poly(dT)-adaptor and reverse transcription. A 2 ug RNA template was used for poly(A)-tail synthesis in the presence of 1.5 units of poly(A)-polymerase, 10X poly(A)-buffer, and 10X ATP in a reaction volume of 15 ul incubated at 37°C for 30 minutes. Annealing of a universal poly(dT)-adaptor was performed at 60°C for 5 minutes. Reverse transcription was carried out by means of iScript™ cDNA Synthesis Kit (Bio-Rad, Hercules, CA, USA), following the manufacturer’s manual. Quantitative real-time RT-PCR (qRT-PCR) was carried out using the CFX Connect Real-Time System (Bio-Rad) and iQ SYBR green supermix (Bio-Rad). Primers’ sequences for qRT-PCR were obtained from the online database miRbase (http://www.mirbase.org/); Table 1 lists the primers’ sequences.

**Table 1:** Sequence of qRT-PCR primers from the miRbase database (www.miRbase.org).

miRNA	Primer Sequence
Universal primer	GCGAGCACAGAATTAATACGAC
miR-191	CAACGGAATCCCAAAAGCAGCTG
miR-21	TAGCTTATCAGACTGATGTTGA
miR-133b	TTTGGTCCCCTTCAACCAGCTA
miR-205	TCCTTCATTCCACCGGAGTCTG
miR-203	GTGAAATGTTTAGGACCACTAG
miR-200a	TAACACTGTCTGGTAACGATGT
mir-194	TGTAACAGCAACTCCATGTGGA
mir-192	CTGACCTATGAATTGACAGCC
mir-31	AGGCAAGATGCTGGCATAGCT

Normalization was performed using the miR-191 as a reference gene. miR-191 has been recognized as stably and consistently expressed among esophageal tissues [15] and is more consistent than other reference RNAs in miRNA qRT-PCR experiments such as 5S rRNA, U6 snRNA, or total RNA [16]. FC was calculated based on the formula 2^(Rt-Et)^, where Rt is the threshold cycle number of the reference gene in the sample. Et is the threshold cycle number of the experimental gene in the sample [17]. The Student’s t-test was used for the evaluation of statistical significance between each two histological groups. A heatmap representing the relative FC of the tumor and normal tissues was constructed by means of Treeview® software [18]. All values were log transformed and centered to the median. Analysis of Variance (ANOVA) was employed for analyzing the difference in miRNA expression among EAC stages I, II, and III. For both Student’s t-test and ANOVA, a p value ≤0.05 was considered statistically significant.

## Results

### Identification and validation of deregulated miRNAs in EAC

**Figure 1:**
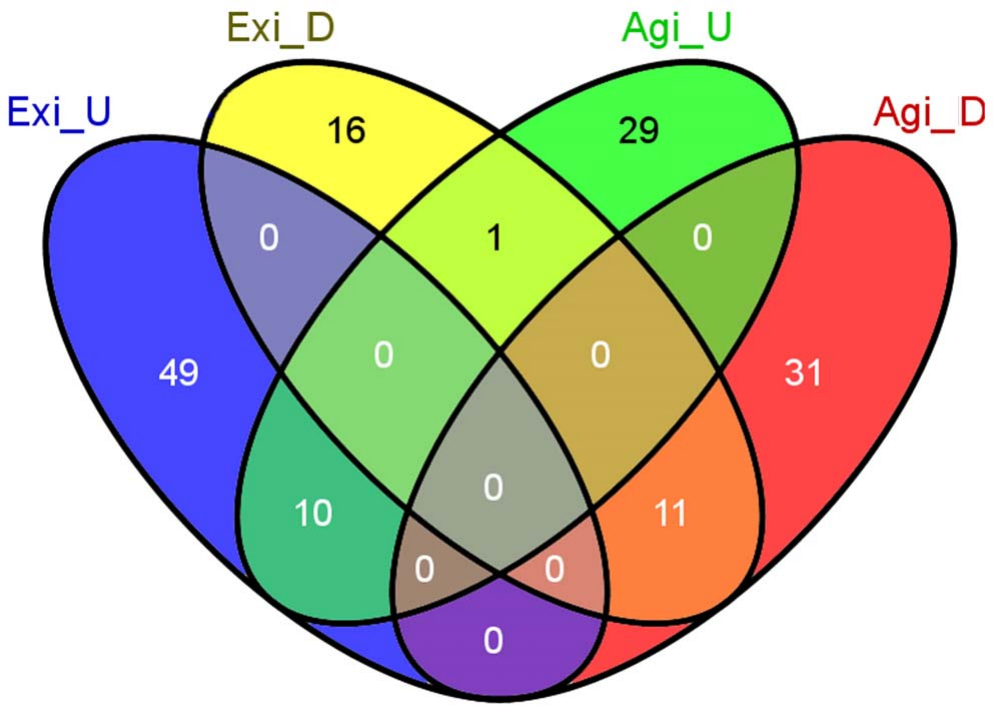
Venn diagram of miRNA analysis in EAC. The Venn diagram was used to identify overlapping and non-overlapping miRNAs in the analysis of Exiqon and Agilent microarrays. 3 EAC tissue samples were used for microarray. 3 pooled NS tissue samples were used as a tissue comparator. miRNAs with ≥2 FC difference (up or down) were included. Exi, Exiqon; Agi, Agilent; D, down-regulated; U, up-regulated.

**Figure 2:**
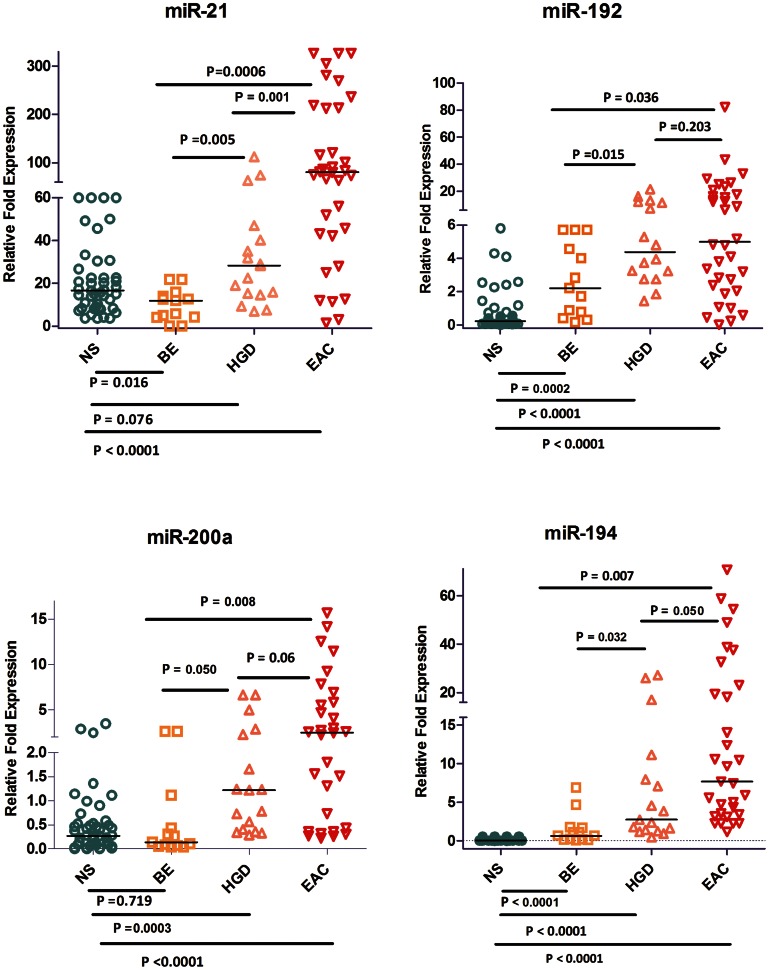
qRT-PCR validation of up-regulated miRNAs in EAC. The expression of 4 miRNAs (miR-192, miR-194, miR-21, and miR-200a) was evaluated using qRT-PCR in 46 NS, 13 BE, 17 HGD, and 34 EAC tissues. The data are plotted as the FC of expression relative to miR-191 expression. Each data point represents a different sample. The horizontal bars indicate the median. Student’s t-test was used for statistical analysis. A value of p≤0.05 was considered statistically significant.

**Figure 3:**
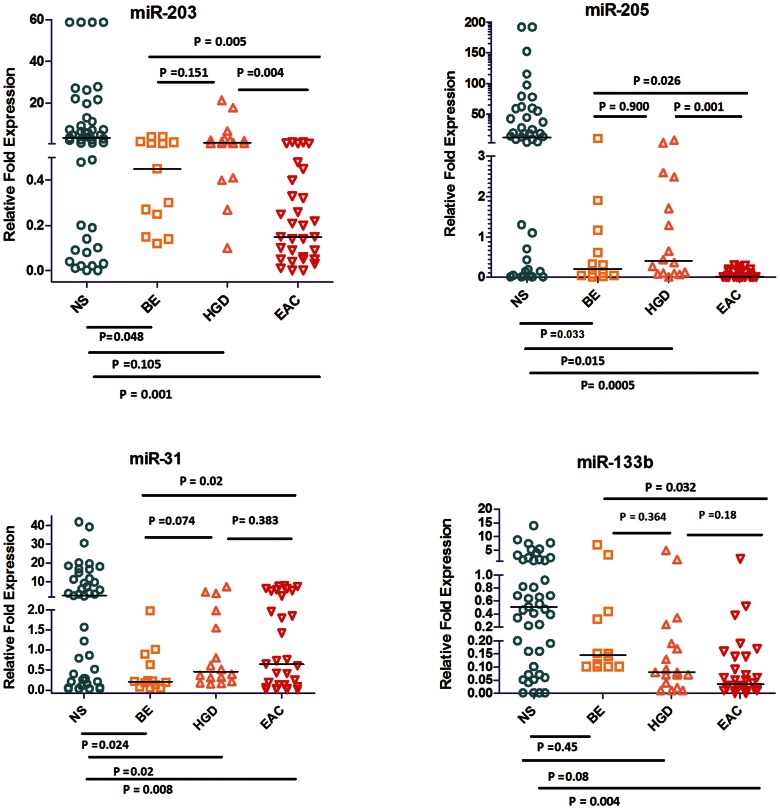
qRT-PCR validation of down-regulated miRNAs in EAC. The expression of 4 miRNAs (miR-205, miR-133b, miR-203, and miR-31) was evaluated using qRT-PCR in 46 NS, 13 BE, 17 HGD, and 34 EAC tissues. The data are plotted as the FC of expression relative to miR-191 expression. Each data point represents a different patient tissue sample. The horizontal bars indicate the median. Student’s t-test was used for statistical analysis. A value of p≤0.05 was considered statistically significant.

**Figure 4:**
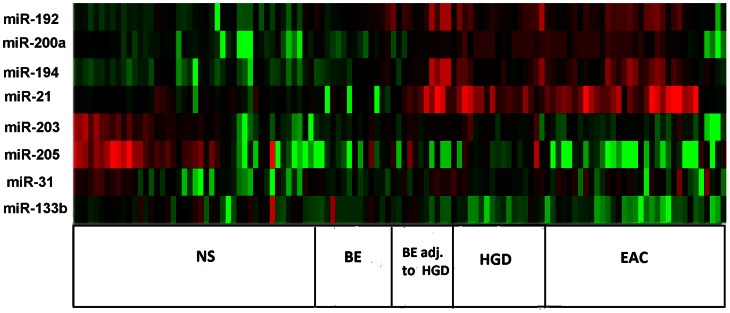
Heatmap of the miRNA signature in EAC. The heatmap was generated using the Treeview® software [18]. The log10 values of fold expression of the 8 miRNAs (miR-192, miR-200a, miR-194, miR-21, miR-203, miR-205, miR-31, and miR-133b) were used for hierarchical clustering. miR-191 was used as a reference gene. The heatmap indicates the four histological groups of NS, BE (isolated BE or BE adjacent to HGD), HGD, and EAC.

Upon comparison of EAC (n = 3) to a pool of 3 adjacent NS tissue samples, we identified 21 miRNAs that are commonly deregulated in both microarray platforms (FC≥2.0 or ≤−2.0, p<0.05); 11 miRNAs were down-regulated and 10 were up-regulated (Figure 1, Table 2). Eight miRNAs that were overlapping between both platforms were subject to subsequent validation by qRT-PCR. We found that 2 miRNAs (miR-192, and miR-194) were up-regulated and 3 miRNAs (miR-205, miR-203, and miR-31) were down-regulated in BE as compared to NS (Figures 2 and 3). We showed the up-regulation of miR-194, miR-192, miR-21, miR-31, and miR-200a (Figure 2, Table 3) and the down-regulation of miR-133b, miR-203, and miR-205 in EAC as compared to BE (Figure 3, Table 3). Our validation confirmed the up-regulation of miR-194, miR-192, miR-21, and miR-200a, and the down-regulation of miR-203, miR-205, miR-133b, and miR-31 in EAC as compared to NS. Hierarchical clustering of log10 transformed values of relative FC expression shows the signature of the 8 miRNAs in the four histological groups of NS, BE (Isolated BE and BE adjacent to HGD), HGD, and EAC (Figure 4).

**Table 2:** miRNAs that displayed at least 2-FC between EAC and NS in both Agilent and Exiqon microarray platforms.

miRNA	Agilent	Exiqon
***Down-regulated in EAC as compared to NS***		
hsa-miR-1274a	−4.19	−3.63
hsa-miR-1274b	−2.22	−2.52
hsa-miR-133b	−6.70	−3.01
hsa-miR-143*	−5.87	−2.25
hsa-miR-144	−7.83	−8.80
hsa-miR-145	−5.63	−4.19
hsa-miR-145*	−3.18	−4.12
hsa-miR-203	−71.85	−30.44
hsa-miR-205	−149.71	−178.03
hsa-miR-31	−8.77	−8.87
hsa-miR-365	−2.78	−2.07
***Up-regulated in EAC as compared to NS***		
hsa-miR-192	145.21	58.40
hsa-miR-194	105.57	52.86
hsa-miR-196a	2.62	2.86
hsa-miR-200a	4.61	4.79
hsa-miR-200b	2.69	3.80
hsa-miR-21	2.25	2.68
hsa-miR-215	131.78	33.06
hsa-miR-429	3.93	3.11
hsa-miR-574-5p	2.19	2.06
hsa-miR-7	2.98	3.98

**Table 3:** Median levels of miRNA expression in NS, BE, HGD, and EAC.

miRNA	NS	BE		HGD		EAC	
	#Median value	#Median value	*P value	#Median value	**P value	#Median value	**P value
miR-194	0.1	0.67	<0.0001	2.73	0.032	7.68	0.007
miR-192	0.2	2.21	0.0002	4.37	0.015	5.00	0.036
miR-21	16.77	11.95	0.01	28.3	0.005	80.30	0.0006
miR-200a	0.2	0.14	0.719	1.22	0.050	2.5	0.008
miR-205	11.87	0.20	0.03	0.4	0.9	0.03	0.026
miR-203	3.33	0.45	0.05	0.93	0.15	0.15	0.005
miR-133b	0.51	0.14	0.45	0.08	0.36	0.03	0.032
miR-31	2.63	0.21	0.024	0.455	0.074	0.64	0.02

# The median values of FC are shown relative to reference miR-191.

* P value is shown for the comparison to NS.

** P value is shown for the comparison to BE.

### Identification of a unique miRNA signature in EAC

**Figure 5:**
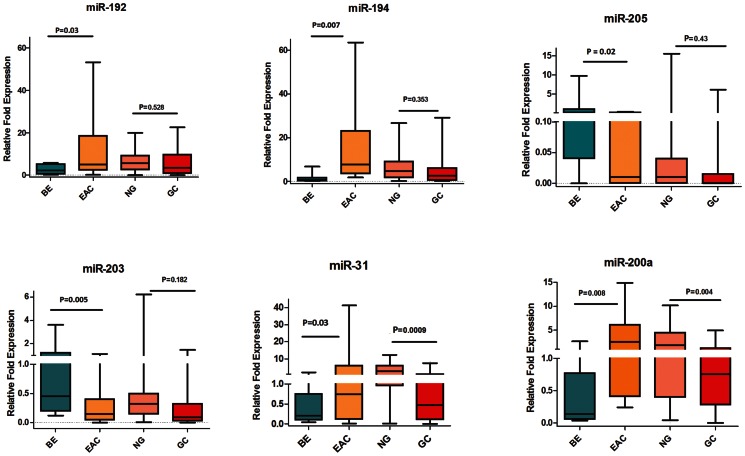
Identification of unique miRNAs for EAC. The expression levels of the 6 miRNAs (miR-194, miR-192, miR-203, miR-205, miR-200a, and miR-31) were measured by means of qRT-PCR in 13 BE, 34 EAC, 45 NG, and 33 GC tissue samples. The FC of expression relative to miR-191 is shown using box-and-whisker plots. The plot groups the data into five categories: the lowest observation, the first quartile (Q1), the median (Q2), the third quartile (Q3), and the largest observation. The horizontal line represents the median value. Data were graphed as a range from 5^th^ to 95^th^ percentile. Student’s t-test was used for statistical analysis. A p value ≤0.05 was considered statistically significant.

**Figure 6:**
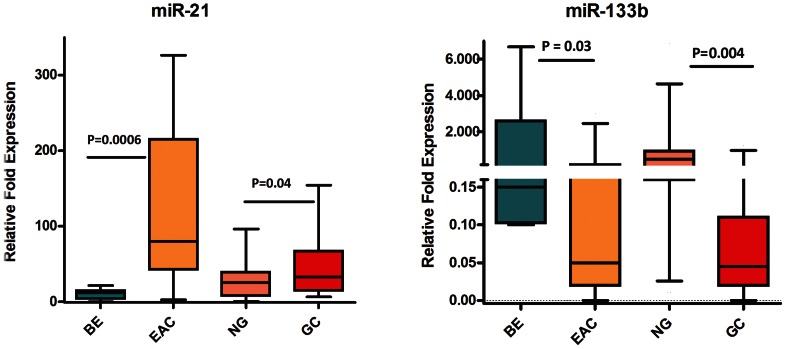
Identification of miRNAs commonly dysregulated in both EAC and GC. The expression levels of the 2 miRNAs (miR-21, and miR-133b) were measured by means of qRT-PCR in 13 BE, 34 EAC, 45 NG, and 33 GC tissue samples. The FC of expression relative to miR-191 is shown using box-and-whisker plots. The horizontal line represents the median value. Data were graphed as a range from 5^th^ to 95^th^ percentile. Student’s t-test was used for statistical analysis. A p value ≤0.05 was considered statistically significant.

We next examined the expression of the 8 miRNAs in GC tissue samples to identify those miRNAs that are unique for EAC. Four miRNAs (miR-194, miR-192, miR-31, and miR-200a) were up-regulated in EAC but not in GC (Table 4, Figure 5). miR-203 and miR-205 were down-regulated in EAC but not in GC (Table 4, Figure 5). These are more likely related to the etiological and biological differences of EAC as opposed to GC. Two miRNAs (miR-21 and miR-133b) were significantly and similarly deregulated in both EAC and GC (Table 4, Figure 6), suggesting that these miRNAs regulate common pathways in UGCs.

**Table 4:** Profile of differentially expressed miRNAs in EAC as opposed to GC.

miRNA	Status in EAC using BE as a comparator	Status in GC using NG as a comparator
miR-194	Up-regulated	Not significant
miR-192	Up-regulated	Not significant
miR-200a	Up-regulated	Down-regulated
miR-31	Up-regulated	Down-regulated
miR-205	Down-regulated	Not significant
miR-203	Down-regulated	Not significant
miR-133b	Down-regulated	Down-regulated
miR-21	Up-regulated	Up-regulated

### Association of the miRNA expression levels with different EAC stages

**Figure 7:**
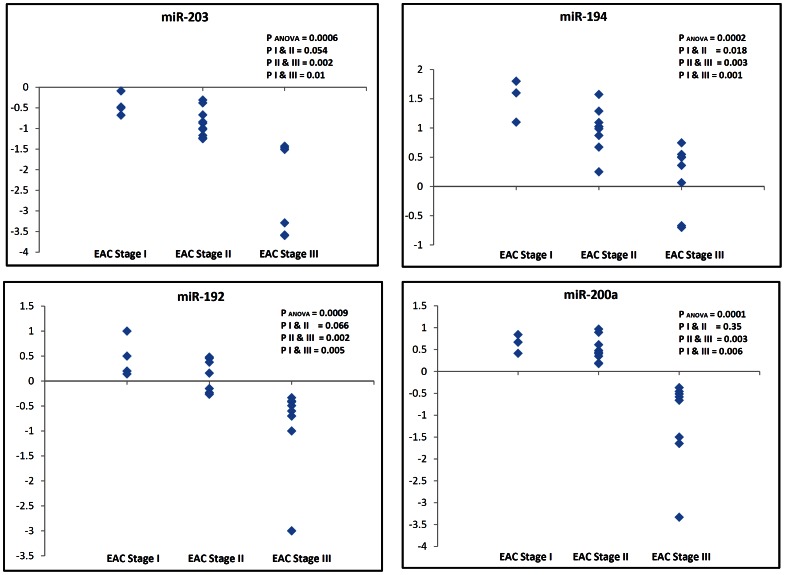
Correlation between the miRNA expression level and EAC tumor stage. The expression levels of the 4 miRNAs (miR-203, miR-194, miR-192, and miR-200a) were evaluated in EAC tissue samples of stages I, II and III by means of qRT-PCR. The data is expressed as the log10 FC relative to miR-191. Each data point represents a different patient tissue sample. ANOVA was used for statistical analysis of the difference in miRNA expression across the 3 stages. Student t-test was used for statistical analysis between stages I&II, stages II&III, and stages I&III. A p value ≤0.05 was considered statistically significant.

To determine if the deregulation of miRNAs plays a role in EAC progression, we evaluated the miRNA expression levels in the various TNM stages of EAC tissues. Samples were stratified from stage I to III based on their TNM classification. The down-regulation of miR-203 was significantly associated with progression and tumor stage in EAC (p_ANOVA_ = 0.0006, p_I&II_ = 0.054, p_II&III_ = 0.002, and p_I&III = _0.01). Of note, the overexpression levels of miR-194, miR-200a and miR-192 were significantly higher in EAC stage I than in advanced stages (P_ANOVA_ ≤0.0009, P_II&III_ ≤0.003, and P_I&III_ ≤0.006), suggesting that these miRNAs may be involved in tumor development rather than tumor progression (Figure 7).

### Differential expression of miRNA between isolated BE and BE lesions adjacent to HGD

**Figure 8:**
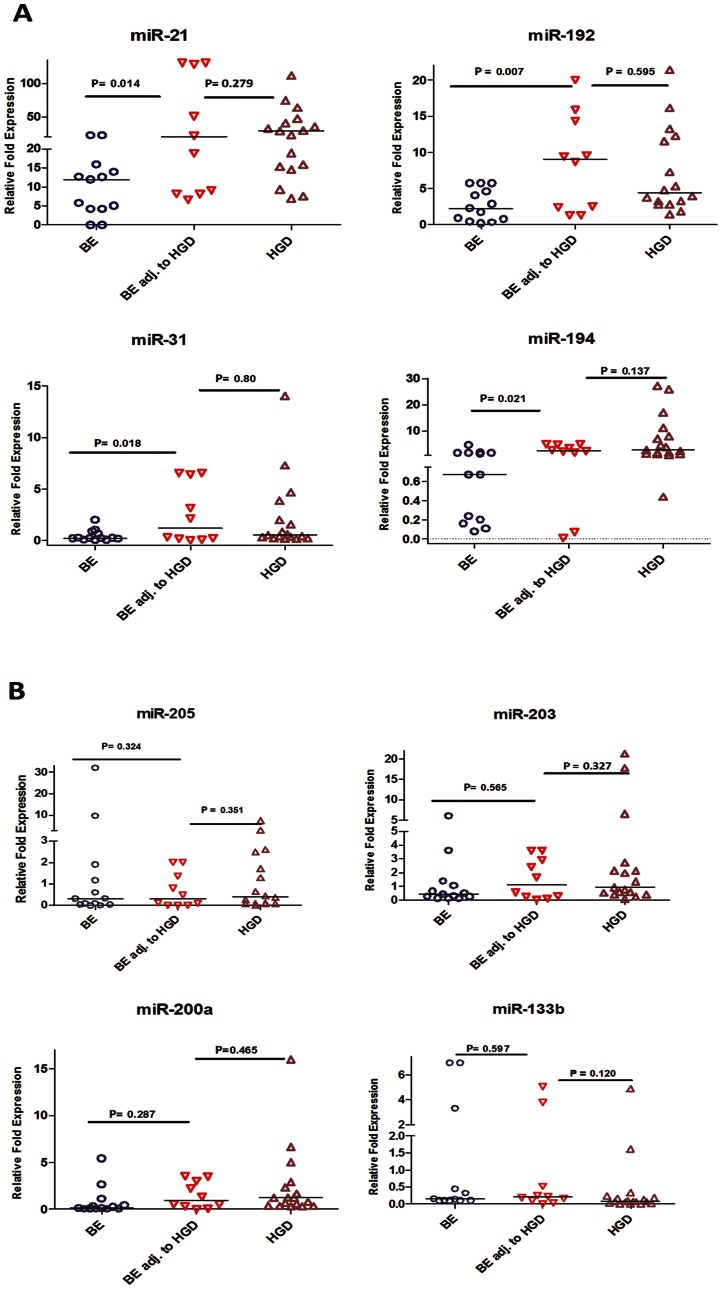
miRNA expression in isolated BE and BE lesions adjacent to HGD. The expression of miRNAs in 13 isolated BE, 10 BE adjacent to HGD, and 17 HGD tissue samples were evaluated by qRT-PCR. The data are plotted as the FC of expression relative to miR-191 expression. Each data point represents a different patient tissue sample. The horizontal bars indicate the median. Student’s t-test was used for statistical analysis. A value of p≤0.05 was considered statistically significant.

We evaluated miRNA expression in 13 isolated BE, 10 BE adjacent to HGD, and 17 HGD samples by qRT-PCR. We found that miR-192, miR-194, miR-31, and miR-21 were significantly up-regulated in BE tissues adjacent to HGD, relative to the isolated BE samples (P<0.05) (Figure 8, Table 5). The BE adjacent to HGD samples expressed these miRNAs at levels comparable to those in HGD samples. On the other hand, miR-133b, miR-200a, miR-205, and miR-203 did not display any significant differential expression between isolated BE and BE adjacent to HGD (Figure 8, Table 5).

**Table 5:** Median levels of miRNA expression in isolated BE and BE adjacent to HGD.

miRNA	Isolated BE	BE adjacent to HGD		HGD	
	# Median value	# Median value	*P value	# Median value	**P value
miR-194	0.67	2.47	0.021	2.73	0.137
miR-192	2.21	9	0.007	4.37	0.595
miR-21	11.95	20.08	0.014	28.3	0.279
miR-31	0.21	1.2	0.018	0.455	0.8
miR-200a	0.14	0.93	0.287	1.22	0.465
miR-205	0.2	0.3	0.324	0.4	0.351
miR-203	0.45	1.12	0.565	0.93	0.327
miR-133b	0.14	0.21	0.597	0.08	0.120

# The median values of FC are shown relative to reference miR-191.

* P value is shown for the comparison to BE.

** P value is shown for the comparison to BE adjacent to HGD.

## Discussion

A considerable body of evidence highlights the aberrant miRNA expression as a key player in the development and progression of gastrointestinal cancers [13], [19], [20]. miRNAs have gained significant attention because of their ability to regulate multiple oncogenic and tumor suppressor signaling pathways. Based on their targets, miRNAs can elicit tumor suppressor or oncogenic actions [8], [9], [11], [21]. This fact placed miRNA in the center of the cancer signaling network that can regulate key biological functions involved in carcinogenesis such as proliferation, invasion, and apoptosis [19], [22]–[24]. In this study, we have employed two different microarray platforms for the preliminary assessment of the miRNAs expression levels in normal and tumor tissues. This approach was employed to identify the most consistent expression changes. The microarray data pointed out the presence of 11 down-regulated and 10 up-regulated miRNAs that were identified in both platforms. The differences between the two platforms could be due to variations in sensitivity of detecting low abundance alterations. Although we acknowledge excluding some miRNAs because of our stringent analytical approach, we presumed that these miRNAs are less likely to be validated or to reflect the biological features of the disease. In fact, all the selected differentially expressed miRNAs identified by this approach were successfully validated by qRT-PCR. We found that the expression of some miRNAs is similar in NS and BE. Other miRNAs displayed a different pattern of expression, which could indicate that they can differentiate between NS and BE. This suggests that these miRNAs could be markers of BE development reflecting the change of the cellular lineage from NS to BE intestinal metaplasia.

Our results indicating up-regulation of miR-21, miR-192, and miR-194 are consistent with previous reports on the miRNA expression profile in EAC [12], [25]. Recent data have suggested that miRNA expression and functions are tissue specific where findings from one disease entity may not be relevant to other ones [10], [11]. While previous reports have shown that EAC and GC have common overlapping features at the molecular level, in studies using CGH or gene expression microarrays [26] there were no direct comparisons investigating miRNAs in both diseases. To address this important question, we have tested the miRNAs that were validated in EAC in a set of gastric tissues. Interestingly, we found that miR-21 (up-regulated) and miR-133b (down-regulated) are similarly deregulated in GC, suggesting that these miRNAs are possibly involved in regulating molecular targets commonly involved in UGCs irrespective of the site and etiology. miR-21 is a well-recognized oncomiR that is up-regulated in almost all epithelial-based solid tumors and is involved in several pathways associated with apoptosis [22]. For instance, miR-21 was shown to target many tumor suppressor genes including the programmed cell death 4 (PCDC4) and TAp63 [22]. Although this is the first time showing deregulation of miR-133b in EAC, its down-regulation was reported in lung cancers. The reconstitution of miR-133b is correlated with reduced expression of MCL1 and BCL2L2, as well as enhanced apoptotic response upon exposure to gemcitabine [24]. In addition, miR-133b sensitizes resistant HeLa and PC3 cells to tumor necrosis factor alpha (TNFα) and to TNF-related apoptosis-inducing ligand (TRAIL) [27].

Using BE as a tissue comparator, we identified 6 miRNAs whose aberrant expression was unique for EAC but not gastric cancer. This is an important finding given the differences in etiology of both diseases as well as the response to therapy and patient outcome. The presence of EAC specific miRNAs suggests that these miRNAs target key molecules involved in signaling pathways associated specifically with EAC. EACs are known to be more resistant to chemotherapeutic regimens and are generally characterized by a poor survival outcome that is worse than is seen in gastric cancers [28]. We demonstrated an inverse correlation between miR-203 expression levels and EAC progression. This finding suggests that miR-203 could be targeting key pathways associated with tumor progression in EAC. Although the targets of miR-203 have not been characterized in EAC, its down-regulation was reported in metastatic breast cancer cell lines and has been linked to increased motility and invasion by targeting SNA12, a transcription factor that enhances invasion and migration [29]. While miR-194, miR-192, and miR-200a were significantly up-regulated in EAC, they also displayed an interesting pattern with disease progression. Their overexpression levels were lower in advanced EAC stages than in early stages. This finding may suggest that these miRNAs are more important in the development of EAC rather than in the progression. The change in the miRNA levels across the stages could also reflect the complexity in cancer signaling where additional molecular hits have been acquired in advanced stages, reducing the need for higher levels of these miRNAs. In addition, our data showed that miR-192, miR-194, miR-21, and miR-31 were significantly dysregulated in BE adjacent to HGD relative to isolated BE tissue samples, showing levels similar to those observed in HGD and EAC. This finding could suggest that these miRNAs may serve as biomarkers for disease progression. However, these findings call for additional investigations to address the diagnostic, functional and signaling components during the progression of EAC.

In summary, this is the first study, to the best of our knowledge, providing a distinction in the miRNA expression profile between EAC and GC. Our findings support the notion that EAC and gastric cancer development and progression are not molecularly identical. Future studies that dissect the targets of these specific miRNAs and their signaling pathways in EAC are expected to provide novel insights into the biology of Barrett’s-related esophageal carcinogenesis.
